# RETRACTION: Recombinant Nanobody Against Muc1 Tandem Repeats Inhibits Growth, Invasion, Metastasis, and Vascularization of Spontaneous Mouse Mammary Tumors

**DOI:** 10.1002/1878-0261.70348

**Published:** 2026-10-20

## Abstract

**RETRACTION:** P. Merikhian, B. Darvishi, N. Jalili, M. R. Esmailinejad, A. S. Khatibi, S. M. Kalbolandi, M. Salehi, M. Mosayebzadeh, M. S. Barough, K. Majidzadeh‐A, F. Yadegari, F. Rahbarizadeh, and L. Farahmand, “Recombinant Nanobody Against Muc1 Tandem Repeats Inhibits Growth, Invasion, Metastasis, and Vascularization of Spontaneous Mouse Mammary Tumors,” *Molecular Oncology* 16, no. 2 (2022): 485‐507, https://doi.org/10.1002/1878‐0261.13123.

The above article, published online on 25 October 2021 in Wiley Online Library (wileyonlinelibrary.com), has been retracted by agreement between the journal Editor in Chief, Kevin Ryan; the Federation of European Biochemical Societies; and John Wiley and Sons Ltd. The retraction has been agreed upon following an investigation into concerns raised by a third party. Specifically, multiple image elements in panels A and B of Figure 6 were found to have been previously published by the author group in a different scientific context. Further investigation by the publisher revealed that Figure 6Ba has been previously published by a different author group in a different scientific context. The clarification and materials provided by the authors were insufficient to address the concerns. The article has been retracted as the editors consider the conclusions to be substantially compromised.

Co‐author B. Darvishi disagreed with the retraction decision. Co‐author F. Rahbarizadeh stated that their contribution was limited to providing the sequence of the nanobody used in the study; co‐author S. M. Kalbolandi stated that their contribution was limited to the production and purification of the recombinant nanobody protein. Both co‐authors F. Rahbarizadeh and S. M. Kalbolandi indicated that they were not involved in the experimental work related to the nanobody's functional characterization nor in the preparation of the related data for the article. No comments on the retraction decision were received from the remaining co‐authors.

**RETRACTION:** P. Merikhian, B. Darvishi, N. Jalili, M. R. Esmailinejad, A. S. Khatibi, S. M. Kalbolandi, M. Salehi, M. Mosayebzadeh, M. S. Barough, K. Majidzadeh‐A, F. Yadegari, F. Rahbarizadeh, and L. Farahmand, “Recombinant Nanobody Against Muc1 Tandem Repeats Inhibits Growth, Invasion, Metastasis, and Vascularization of Spontaneous Mouse Mammary Tumors,” *Molecular Oncology* 16, no. 2 (2022): 485‐507, https://doi.org/10.1002/1878‐0261.13123.

The above article, published online on 25 October 2021 in Wiley Online Library (wileyonlinelibrary.com), has been retracted by agreement between the journal Editor in Chief, Kevin Ryan; the Federation of European Biochemical Societies; and John Wiley and Sons Ltd. The retraction has been agreed upon following an investigation into concerns raised by a third party. Specifically, multiple image elements in panels A and B of Figure 6 were found to have been previously published by the author group in a different scientific context. Further investigation by the publisher revealed that Figure 6Ba has been previously published by a different author group in a different scientific context. The clarification and materials provided by the authors were insufficient to address the concerns. The article has been retracted as the editors consider the conclusions to be substantially compromised.

Co‐author B. Darvishi disagreed with the retraction decision. Co‐author F. Rahbarizadeh stated that their contribution was limited to providing the sequence of the nanobody used in the study; co‐author S. M. Kalbolandi stated that their contribution was limited to the production and purification of the recombinant nanobody protein. Both co‐authors F. Rahbarizadeh and S. M. Kalbolandi indicated that they were not involved in the experimental work related to the nanobody's functional characterization nor in the preparation of the related data for the article. No comments on the retraction decision were received from the remaining co‐authors.

